# Bevacizumab combined with gemcitabine and capecitabine for advanced pancreatic cancer: a phase II study

**DOI:** 10.1038/sj.bjc.6605099

**Published:** 2009-06-02

**Authors:** M Javle, J Yu, C Garrett, A Pande, B Kuvshinoff, A Litwin, J Phelan, J Gibbs, R Iyer

**Affiliations:** 1 Department of Medicine, Roswell Park Cancer Institute, Elm and Carlton Streets, Buffalo, NY 14263, USA; 2 Department of Biostatistics, Roswell Park Cancer Institute, Elm and Carlton Streets, Buffalo, NY 14263, USA; 3 Department of Medical Oncology, H Lee Moffitt Cancer Institute, 12902 Magnolia Drive, Tampa, FL 33612, USA; 4 Department of Surgery, Roswell Park Cancer Institute, Elm and Carlton Streets, Buffalo, NY 14263, USA; 5 Department of Radiology, Roswell Park Cancer Institute, Elm and Carlton Streets, Buffalo, NY 14263, USA; 6 Department of Oncology, Lipson Cancer Center, 1445 Portland Avenue, Rochester, NY 14621, USA

**Keywords:** gemcitabine, capecitabine, bevacizumab, pancreatic cancer, chemotherapy, metastatic

## Abstract

A total of 50 patients with advanced pancreatic cancer were enrolled in a phase II study of bevacizumab 15 mg kg^−1^, capecitabine 1300 mg m^−2^ daily for 2 weeks and gemcitabine 1000 mg m^−2^ weekly 2 times; cycles were repeated every 21 days. Radiological response rate was 22%; progression-free survival and over survival were 5.8 and 9.8 months respectively. Grade 3 or 4 toxicities included neutropaenia (22%), thrombocytopaenia (14%), thromboembolic events (12%), hypertension (8%) and haemorrhage (6%).

Metastatic pancreatic cancer is one of the most chemotherapy-resistant tumours. Gemcitabine is the chemotherapeutic agent of choice. However, gemcitabine results in a clinical benefit response rate (RR) of 24% and a 1-year survival of 18% only for patients with advanced pancreatic cancer (Burris III *et al*, 1997). The addition of other cytotoxic agents to gemcitabine including capecitabine, cisplatin, irinotecan and oxaliplatin does not lead to any improvement in overall survival (OS) (Rocha Lima *et al*, 2004; Louvet *et al*, 2005; Heinemann *et al*, 2006; Herrmann *et al*, 2007). However, gemcitabine-based combinations may have value in patients with good performance status (PS). Recently, Herrmann *et al* (2007) reported that pancreatic cancer patients with good PS may experience improved OS and progression-free survival (PFS) with gemcitabine+capecitabine as compared with gemcitabine alone. The vascular endothelial growth factor (VEGF) is a potent angiogenic factor and represents a therapeutic target in pancreatic cancer (Ferrara *et al*, 2003). Increased VEGF expression occurs in most human tumours including pancreatic cancer (Yoshiji *et al*, 1996; Soh *et al*, 2000; Huang *et al*, 2001; Deryugina *et al*, 2002; Bremnes *et al*, 2006; Ozdemir *et al*, 2006; Black and Dinney, 2007). Bevacizumab (rhuMAb VEGF) is a recombinant humanised anti-human VEGF monoclonal antibody, which results in a synergistic anti-tumour effect in preclinical studies when combined with fluoropyrimidines or gemcitabine (Margolin *et al*, 2001; az-Rubio and Schmoll, 2005; Kindler *et al*, 2005a). The present study explored the clinical activity of gemcitabine, capecitabine and bevacizumab in patients with advanced pancreatic cancer.

## Patient eligibility

All patients provided written informed consent before study enrollment. Adult patients with previously untreated metastatic or locally advanced unresectable pancreatic cancer, Eastern Cooperative Oncology Group (ECOG) PS of 0 or 1, normal blood counts (leucocytes>3000 per *μ*l, neutrophils>1500 per *μ*l, platelets>100 000 per *μ*l) and chemistries (bilirubin<2 mg per 100 ml, AST/ALT<5 times upper limits of normal, creatinine<1.5 mg per 100 ml) were included. Prior adjuvant therapy was permitted if completed >6 months before enrollment. Exclusion criteria included proteinuria, pregnancy, lactation, bleeding diathesis, uncontrolled hypertension or cardiovascular disease, brain metastases or recent surgery.

## Treatment plan

Gemcitabine was administered in a dose of 1000 mg m^−2^ intravenously over 30 min on days 1 and 8; capecitabine 650 mg m^−2^ twice daily was administered on days 1–14 and bevacizumab 15 mg kg^−1^ was administered after gemcitabine on day 1 of a 21-day cycle. Treatment was continued until disease progression, death or toxicity. A maximum of 1 year of bevacizumab therapy was permitted. However, patients could receive gemcitabine and capecitabine beyond 1 year if indicated. Institutional review board approval was obtained for this study.

## Dose adjustments

Dose reductions for gemcitabine and capecitabine were based on manufacturer guidelines. Adverse events were graded according to National Cancer Institute, Common Toxicity Criteria, version 3.0 (NCI-CTC v 3.0). A cycle was not started until the absolute neutrophil count was >1500 per *μ*l and platelet count was >100 000 per *μ*l. Dose adjustments for gemcitabine were based on the laboratory and clinical findings on the scheduled day of administration, whereas the dose adjustment of capecitabine was based on the toxicities during the preceding cycle. There were no dose adjustments for bevacizumab in this study. Bevacizumab was held for grade 3 hypertension, grade 3 thrombosis, grade 3 haemorrhage or proteinuria ⩾2 g, until resolution. Bevacizumab was permanently discontinued for grade 4 or recurrent grade 3 vascular events. Routine use of neutrophilic growth factors was not recommended.

## Study evaluations

Pretreatment included complete history and physical exam, complete blood count, chemistry including liver function tests, prothrombin time, pregnancy test for women and 12-lead electrocardiography. Urine protein/creatinine ratio was measured at baseline and every 6 weeks. History and physical exam were performed every 3 weeks. Complete blood count, serum CA 19-9 level and serum chemistries (including liver function tests) were measured on day 1 of each treatment cycle. Computed tomography scans to assess tumour size and response were obtained every 6 weeks.

The PFS was defined as the length of time during and after treatment in which the patient remained alive with cancer without disease progression. Overall survival was defined as the time from treatment initiation until demise. Responses were estimated using the response evaluation criteria in solid tumors (RECIST) (Therasse *et al*, 2000). CA 19-9 improvement by 50% was defined as a ‘CA 19-9 response’.

## Statistics

The primary study aim was evaluation of PFS with the combination therapy for patients with pancreatic cancer. Secondary aims were estimation of RR, toxicity and OS. We calculated 95% confidence intervals for estimated PFS and OS curves using the Greenwood formula. The sample size was calculated to provide estimations of median PFS and median OS with reasonable accuracy. The projected 95% confidence interval width with 50 patients was approximately 3.5 months. The survival curves for OS and PFS were estimated by the Kaplan–Meier method. The Clopper–Pearson method was used to estimate the 95% confidence interval for the RR.

The association of survival and quantifiable variables, including age, grade and CA 19-9 level, was univariately investigated using the log-rank test.

## Results

### Patient characteristics

A total of 50 patients from three institutions were enrolled in this study between 7 September 2004 and 3 March 2007. The median follow-up duration was 8.9 months. Patient characteristics are listed in Table 1.

### Treatment administration

A total of 348 cycles were administered. Median number of cycles delivered was 6 (range, 1–18). Dose modification for toxicities was required in 25 (50%) of patients. Gemcitabine dose was reduced in 20 (40%) and capecitabine in 13 (26%) patients. Reasons for treatment discontinuation are described in Table 2.

### Toxicities

Haematological toxicities were common (Table 3). There were no cases of febrile neutropaenia. Grade 3 or 4 non-haematologic toxicities included two cases of diarrhoea and hand–foot syndrome secondary to capecitabine and two cases with liver function abnormalities, most likely related to biliary stent occlusion. Bevacizumab-related toxicities were hypertension, haemorrhage and thrombosis (Table 4). Most of the bleeding events were grade 1 or 2 in severity and included epistaxis (*n*=3) or lower gastrointestinal bleeding events (*n*=3). There were three cases of grade 3 haemorrhage, all of which were gastrointestinal. There was one case of grade 5 haemorrhage. This patient had cancer involvement of the gastric wall and varices. Subsequently, the study was amended: all patients with gastric involvement or varices were excluded. There were no subsequent grade 5 toxicities.

### Efficacy

**Figure 1:**
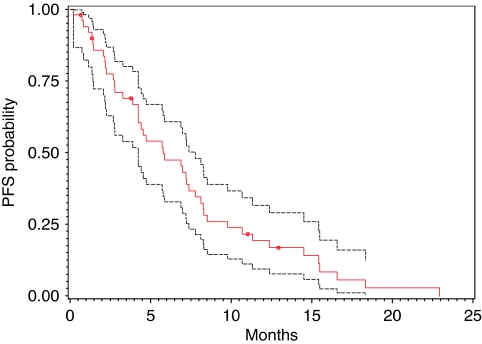
PFS curve (red) with 95% CI (black).

**Figure 2:**
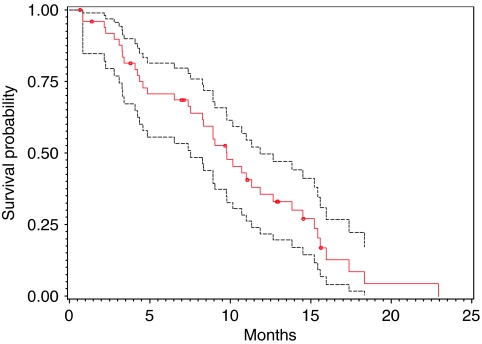
OS curve (red) with 95% CI (black).

All 50 patients were included in an intention-to-treat survival and response analysis. The radiological responses were independently confirmed by the Response Review Committee. A 22% RR (PR+CR) was recorded in this trial. The median PFS was 5.8 months and the median OS was 9.8 months (Figure 1 and 2; Table 5).

A 50% decline in CA 19-9 levels was seen in a larger number of patients (65%). There was a statistically significant correlation between 50% CA 19-9 decline and PFS (*P*<0.0001, log-rank test), OS (*P*=0.0008, log-rank test) and response (*P*=0.0069, exact *χ*^2^-test).

## Discussion

The majority of patients with pancreatic cancer have metastatic disease at diagnosis and their survival has not significantly changed over the past two decades (Baxter *et al*, 2007). Patients who have a poor PS derive a marginal benefit from systemic chemotherapy. The addition of bevacizumab to this combination was based on preclinical and clinical data available at the time this study was instituted (Chen, 2004; Kindler *et al*, 2005b). The dosage of bevacizumab used was 15 mg kg^−1^, which was proven as effective in combination with systemic chemotherapy for non-small-cell lung cancer (Sandler *et al*, 2006). The bevacizumab-related toxicities noted in this study were manageable and similar to those reported at lower doses of this agent (Hurwitz and Saini, 2006). The study permitted administration of bevacizumab for a maximum of 12 months as there was no safety data beyond that duration. CA 19-9 decline was a useful surrogate marker for response, PFS and OS in the present study. Similar results have been reported by others (Halm *et al*, 2000; Ko *et al*, 2005). We used PFS as the primary study end point. Overall survival and RR are more commonly used. In our study, imaging studies were performed at 6-week intervals, adding to the robustness of the PFS data. In a recent meta-analysis, improved PFS and not RR correlated with an improvement in OS (Bria *et al*, 2007). Furthermore, OS may be confounded by second-line therapy.

The Cancer and Leukemia Group B (CALGB) randomised phase III study of gemcitabine±bevacizumab reported no survival advantage with the addition of bevacizumab (Kindler *et al*, 2007). Another recent, randomised phase III study of gemcitabine, erlotinib±bevacizumab for advanced pancreatic cancer did not meet its primary end point of improved survival in the bevacizumab arm (Van Cutsem *et al*, 2009). Based on the results of our study and the above two studies, the role of bevacizumab therapy in this disease appears to be questionable and we are not proceeding with a phase III study. This does not however reflect the role of anti-angiogenic strategies in pancreatic cancer, which are worthy of further study.

We conclude that the combination of gemcitabine, capecitabine and bevacizumab is active in pancreatic cancer. Future investigational strategies should focus on the identification of subgroups that may benefit from the addition of anti-angiogenic therapy for pancreatic cancer.

## Figures and Tables

**Table 1:** Patient characteristics

**Characteristic**	**Number**	**Range**	**Percentage**
Total	50	—	100
Median age (years)	64	38–83	—
Sex: males	28	—	56
Stage:			
locally advanced	3	—	6
metastatic	47	—	94
Prior surgical resection and adjuvant therapy	5	—	10
Median CA 19-9 level	963 U ml^−1^	9.3–1 089 979 U ml^−1^	
Sites of metastases (measurable):			
Liver	28	—	—
Distant lymph node	2	—	—
Lung	4	—	—
Intra-abdominal	11	—	—

**Table 2:** Reasons for treatment discontinuation

**Reason**	**Frequency (%)**
Completed 1 year of bevacizumab	1 (2)
Disease progression	24 (48)
Toxicity	18 (36)
Death while on treatment	4 (8)
Other^a^	3 (6)

a One had symptomatic deterioration, one had open wounds and one was at discretion of investigator.

**Table 3:** Treatment-related grade 3 or 4 toxicities

**Toxicity**	**Grade 3**	**Grade 4**
*Hematologic*		
Anaemia	3	0
Neutropaenia	11	0
Thrombocytopaenia	5	2
		
*Non-hematologic*		
Fever	1	0
Fatigue	1	1
Diarrhoea	2	0
Hand–foot syndrome	2	0
Liver enzyme elevation (AST/ ALT)	4	0
Oedema	2	0

**Table 4:** Adverse events possibly related to bevacizumab

**Toxicity**	**Grade 1**	**Grade 2**	**Grade 3**	**Grade 4**
Hypertension	6	6	4	0
Proteinuria	0	1	1	0
Haemorrhage	3	4	3	0
Thrombosis	0	2	3	0
Headache	3	1	1	0
Myocardial infarct	0	0	0	1

One patient had grade 5 haemorrhage.

**Table 5:** Clinical efficacy data

**Efficacy parameter**	**Value**	**95% CI**
*Radiologic responses*		
Partial response	10 (20%)	—
Complete response	1 (2%)	—
Stable disease	30 (60%)	—
Progressive disease	5 (10%)	—
Not evaluable^a^	4 (8%)	—
		
PFS	5.8 months	4.2–7.8 months
OS	9.8 months	8.3–11.9 months
1-year survival	35.5%	21.7–49.5%
1-year PFS	19%	9.4–31.6%
		
*CA 19-9*		
50% improvement	31 (65%)	0.49–0.78
Median duration of improvement.	4.46 months	—

PFS=progression-free survival; OS=overall survival.

a Not evaluable due to disease progression clinically.
